# An Ultra-Area-Efficient 1024-Point In-Memory FFT Processor

**DOI:** 10.3390/mi10080509

**Published:** 2019-07-31

**Authors:** Hasan Erdem Yantir, Wenzhe Guo, Ahmed M. Eltawil, Fadi J. Kurdahi, Khaled Nabil Salama

**Affiliations:** 1 Sensors Lab, Advanced Membranes & Porous Materials Center (AMPMC), Computer, Electrical and Mathematical Sciences and Engineering Division, King Abdullah University of Science and Technology (KAUST), Thuwal 23955-6900, Saudi Arabia; 2 Center for Embedded and Cyber-physical Systems, University of California, Irvine, CA 92697, USA

**Keywords:** Fast Fourier Transform, in-memory computing, associative processor, non-von neumann architecture

## Abstract

Current computation architectures rely on more processor-centric design principles. On the other hand, the inevitable increase in the amount of data that applications need forces researchers to design novel processor architectures that are more data-centric. By following this principle, this study proposes an area-efficient Fast Fourier Transform (FFT) processor through in-memory computing. The proposed architecture occupies the smallest footprint of around 0.1 ${\mathrm{mm}}^{2}$ inside its class together with acceptable power efficiency. According to the results, the processor exhibits the highest area efficiency (FFT/s/area) among the existing FFT processors in the current literature.

## 1. Introduction

Today’s processor-centric design principle of computer architectures causes a great deal of energy waste. This is mainly because processing on the data is performed far away from the data [1]. Moreover, even though the processor systems are highly optimized, the data units are not considered much. On the other hand, computer applications are becoming increasingly data hungry. This became an indispensable fact especially after the rise of artificial intelligence (AI) and deep-learning domains for which big data is necessary [2]. Therefore, data movement energy dominates to compute energy in a traditional computer architecture serving today’s computational needs. For example, memory access nearly consumes 1000× the energy of a complex addition operation [3]. Since the amount of data required increases, this adversely affects the efficiency of computers. Not only for AI but also in all domains ranging from signal processing to robotics, an efficient and memory-optimized computation is desired for the sake of specific advantages. Therefore, this fact forces researchers to find alternative computation methodologies. A paradigm shift to perform the computation with minimal data movement is needed by computer scientists. The most reasonable way to achieve this is by making the computation more data-centric than at present processor-centric. This research goal is investigated by many different methodologies. In the ideal case, the most advantageous computing methodology is in-memory computing means that data is processed where it resides.

In-memory computing can be achieved through different methodologies [4]. The most straightforward method is placing memory and processor inside the same chip to facilitate ultra-fast data processing instead of moving the data through the slow buses between the different chips [5]. Even though the idea seems as simple, this combination requires special fabrication in-chip manufacturing. The architecture targets to combine the processor logic with a stack of through-silicon-via (TSV) bonded memory die [6,7]. The logical core of the memory system is a kind of single instruction multiple data (SIMD) processor where the different memory portions are directly connected to different cores, thus increasing the overall system bandwidth. As another methodology, some researchers focus to insert extra abilities to existing memory chips with minimal modifications. As a basic motivation example, 5% of the overall cycles in Google’s data centers are spent on memcopy and memmove operations [8]. If a dynamic random-access memory (DRAM) has the capability to exchange data between its rows without processor intervention, then these operations would not have to be carried over the central processing unit (CPU). The study in [9] modifies the DRAM chip to perform this operation directly inside the DRAM without moving the data. The modification increases the DRAM area only 0.01%. Emergence of the new nonvolatile memory (NVM) technologies such as resistive RAM (ReRAM) and phase change memory (PCM) created a widespread adaptation for in-memory processing due to their inherently analog processing capability, high density, and scalability [10,11]. There are many studies that aim to perform in-memory computation by using NVMs, but with different methodologies [12,13]. An example of this kind in-memory computation methods is using memristor crossbars where the crossbar is configured in a way to perform corresponding specific operations. When an input is applied to the programmed crossbar, its corresponding output becomes the result of the programmed operation [14]. The study in [15] exploits the memristor crossbar for approximate addition and multiplication operations. The prime architecture proposed in [16] uses memristor crossbars to create a neural network realization which is the fundamental operation in deep learning. Another approach of in-memory computing is integrating simple logic structures in each memory cell [17]. The study in [18] proposes an architecture in which the memory cells can both store the data and perform simple computations on it. Furthermore, two or more cells can be combined to perform more complex operations. Another study in [19] proposes a systolic three-layer memory structure consists of memory, routing, and logic planes.

As another methodology, associative in-memory processing performs the in-memory computation by using look-up tables of the arithmetic and logical operations. Unlike the traditional von Neuman or near-memory computation in which the data sent to a processor for computation, associative processors (AP) sent the functionality (i.e., operation) over the data without moving it. In other words, functionality is performed directly inside the memory. Table 1 summarizes the comparison between these methodologies. According to the specifications, in-memory processing provides the broadest constraint in terms of bandwidth. With the invention of resistive memory devices such as ReRAM [20], STT-RAM (spin-transfer torque random-access memory) [21] this convention has started to gain popularity recently. Since there are numerous studies performing in-memory computation through different approaches, the study in [18] puts an extra effort for the taxonomy and proposes a classification into four groups; computation-near-memory (processor and memory in the same chip), computation-in-memory (computation is performed in the peripheral circuitry of the memory), computation-with-memory (LUTs are used for computation), and logic-in-memory (the memory cells have the computation ability). Regarding this classification, associative processing can be considered to be a computation-with-memory approach.

In this study, a fast and efficient in-memory accelerator/processor is proposed for the Fast Fourier Transform (FFT) which is the most important and extensively used algorithm in signal processing. Since the computation domain has already reached to big data era, signal processing architectures should be reconsidered from the data perspective. As a supportive case from health industry, magnetic resonance imaging (MRI) requires huge data sampling and processing for better patient diagnosis. Fast Fourier Transform is heavily used during the processing [22]. If the computation is inadequate in performing the FFT at enough speed, the patient must stay longer inside the MRI [23,24], therefore be exposed to more stress. On the contrary, if the sampled signal size is decreased, the accuracy is affected negatively which is not acceptable in the health industry. Therefore, a fast FFT processor is required to acquire both enough accuracy and processing speed. The proposed architecture exploits the different FFT computation methodologies which have a coherence inherently for in-memory computing to come up with the efficient architectures. The study also proposes the overall integration solution in which accelerator can be used as a standalone processor on its own.

The rest of the paper is organized as follows: In the following section, the fundamental idea of associative computing together with the architecture is presented. Section 3 introduces the proposed two architectures of in-memory FFT processor that are throughput-optimized and area-optimized, respectively. Experimentation and evaluation results are discussed in Section 4. The final section concludes the work.

## 2. In-Memory Associative Processing

Associative in-memory processing is a computing paradigm aims to perform the operations on the data by using associativity principles [25]. The proposed FFT processor in this study bases the associative in-memory processing. All primitive FFT operations are performed on the input data placed inside the memory without moving it. The following two subsections form a background on the AP architecture as well as how associative computing is performed.

### 2.1. Associative Computing

**Figure 1:**
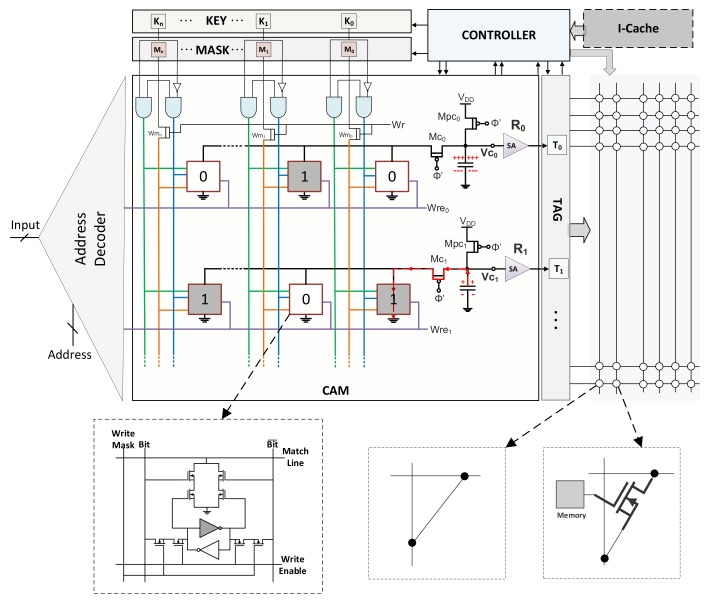
Associative processor architecture.

Figure 1 shows the overall architecture of an AP in detail. The key component of an AP is a content addressable memory (CAM) [26,27]. A CAM is used to access the data by its content on the contrary to the traditional memory where the data is accessed by its address. The CAM stores the data on which the operations are performed. The figure shows the SRAM-based CAM cell structure. In this cell, the one-bit data is stored by a coupled inverter where each inverter supports to the other to keep its logical value. Associative processing exploits the associativity feature of the CAMs hence the name comes from. The basic operation on a CAM is done through the *key*, *mask*, and *tag* registers which are managed by the *controller*. A search operation inside the CAM can be performed as follows; First, the content which searched for inside the CAM is written to the key. The mask register identifies the columns on which the search is performed. If the content is found in a row, the corresponding tag register of this row becomes logic-1.

In addition to CAM, AP needs an address decoder for the communication with the outer system. This outer system can directly be a data source or a processor. Depending on the usage, AP can function as either a standalone processor or an accelerator. The computation inside the CAM is performed in a SIMD fashion. On the other hand, the traditional processors or outer systems (e.g., sensors) provide the data as sequential. To interact between these two different systems, an address decoder is used to feed or output data as sequential by activating the specific rows of the CAM.

As detailed in the next subsection, APs are very powerful for performing parallel operations when the provided data is on the same row. On the other hand, if the benchmark requires computation not only as pairwise (e.g., vector dot product) but also between the different pairs (e.g., matrix multiplication), it needs data exchange between the rows. For this purpose, a *switching matrix* is used to move the data as column-wise between the APs or to the same AP. This communication must be configurable if the processor supports different kinds of tasks with different communication patterns. On the other hand, if the processors is an application specific, it can be fixed. Figure 1 shows these two kinds of approaches in the interconnection matrix.

### 2.2. Operation

The main idea of associative in-memory processing is performing the function/operation on the data without moving it. In traditional processors, to perform an operation on a set of data, the data is moved to the processor through the special high-speed buses and brought to the processor. Inside the processor, the data passes through the functionality (e.g., a full adder or multiplier) and the computed results are written back to the memory. Unlike this approach of sending data over functionality, in in-memory associative processing, the functionality is sent over the data (see Table 1). Even though this approach seems unconventional, the CAM structure inside the AP makes it feasible.

The operations on the AP are performed through the compare and write cycles. During the compare cycle, a specific key (data) is searched for inside the CAM and in the write cycle, the specific data can be written to the columns which have the searched content (i.e., matched as a result of compare operation). Since a specific content can be selected in the CAM through the compare cycles, the corresponding function on this specific content can be applied to data inside the CAM. As an example, to perform the logical NOT operation (i.e., B = ∼A where column B is initialized with logic-0), the CAM is searched for logic-0 on the input column (i.e., column A) and a logic-1 is written to the column B of the matched rows. Therefore, the logical not operation can be applied to the data which is logic-0. In the end, the rows with logic-1 in Column A have logic-0 in Column B and vice versa. Therefore, by applying the special functionality with respect to the searched content, the intended function can be performed. The functionalities of the AP operations are defined by look-up tables (LUTs). Depending on the LUT, the corresponding functionality is applied to the rows of the CAM separately. Table 2 shows two example LUTs for in-place addition (i.e., B←B+A) and subtraction (i.e., B←B−A) operations where Cr and Br are stand for carry and borrow respectively. The operations are performed as bitwise, starting from the least significant bit (LSB) of the operand towards the most significant bit (MSB). On each bit, the LUT passes are applied through the compare and write cycles. As an example of addition operation, in the first LUT pass, “011” is searched for in the CAM array for Cr, B, and A bits respectively during the compare cycle and then “10” is written to the Cr and B columns of the matched rows. The entries of LUT are iteratively applied to all bits of B and A in sequence by following the order. The comment column indicates the order that LUT entries are applied required to perform the operation correctly. Some LUT entries are unnecessary and do not participate in the result; therefore, they are indicated as NC (no change) in the comment column. The studies in [27,28,29], show the detailed examples of some arithmetic and logical AP operations in detail together with the step-by-step illustrations.

## 3. FFT Processor Architecture

The Fourier transform is a function used to decompose the given signals into its sinusoidal components [30]. It is used in nearly all scientific domains ranging from signal processing to artificial intelligence. In 1965, Cooley and Tukey proposed a faster algorithm named FFT to compute the Discrete Fourier Transform (DFT) [31] where the complexity of the transform decreased to $O(n\log_{2}n)$ from $O(n^{2})$. The proposed faster methodology consists of the interleaved computation stages where each stage composes of basic butterfly operations performed on data pairs. Since the algorithm is highly parallel, it inherently provides a widespread adaptation for in-memory associative processing both has a computation structure in an SIMD fashion [32]. On the other hand, the architecture requires some modifications to fulfill the requirements of an efficient processing platform. The following subsections detail the proposed implementations of FFT on in-memory AP in a hierarchical manner.

### 3.1. Butterfly Operation

**Figure 2:**
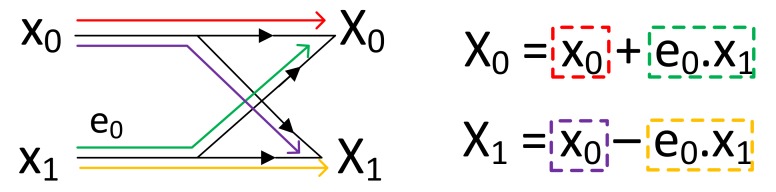
Simple butterfly operation.

The butterfly operation is the fundamental building block of an FFT stage. Figure 2 shows the simplest butterfly diagram consisting of two inputs, two outputs and one exponential coefficient (twiddle factor) where all numbers are complex (i.e., $X_{0},X_{1}=\mathrm{butterfly}\left(e_{0},x_{0},x_{1}\right)$).

**Figure 3:**
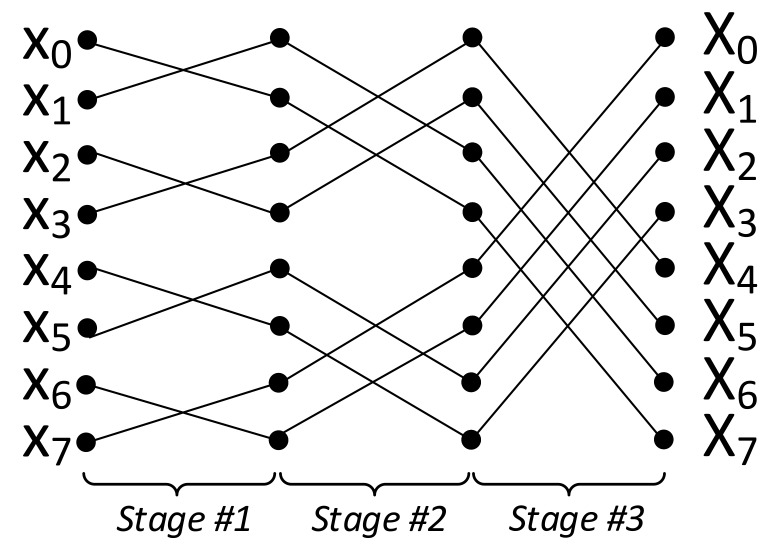
8-point traditional FFT.

Figure 3 shows the data flow of radix-2, decimation in time, 8-point Cooley-Tukey’s FFT in three stages where each stage consists of four butterfly operations. After each stage, the partial outputs of previous stages are rearranged as an input of the next stage. From the AP-based point of view where each row can be regarded as a different processor with their own registers, two input and one exponential factor must be stored in the same row to perform a butterfly operation. However, after completion of a butterfly stage, the output of the current stage must be rearranged for the next stage since the computation pattern changes and the AP can perform the butterfly operation if and only if the operands (i.e., two inputs and coefficient) are in the same row. The exponential coefficients ($e_{xy}$) can be placed to the CAM arrays before the operations. For an n-point FFT operation, the overall system requires $log_{2}\left(n\right)$ APs and each AP requires $\frac{n}{2}$ rows. For example, the system requires 10 APs and 512-rows in each AP for 1024-point FFT operation. Since this is an in-memory FFT processor, the memory requirement is higher than the traditional FFT processors (e.g., [33,34,35]). On the other hand, the proposed processor does not need any traditional logic circuit, therefore provides an overall area efficiency. Section 4 discusses the comparison in detail.

### 3.2. Data Movement

To process the data inside the AP accelerator, the outer system (i.e., processor) needs to communicate well enough with the accelerator (i.e., FFT processor). To feed the input data and retrieve the output data, the processor should have access to the data of the CAMs as row addressed. The main reason for this is that the traditional processors process the data as row-wise on the contrary of APs where data is processed as column-wise (see Section 2). Additionally, the sensors sample the data in time as sequential and provide it in this manner. The Figure 1 shows this hierarchy where the address decoder handles the communication between the AP and the processor. This decoder activates the specific row of the AP as described in the address input. The previous studies on associative computing [27,36] also provide a decoder mechanism for this purpose. In such an architecture, every row of the AP becomes addressable by the outer processor. On the other hand, the main purpose of in-memory accelerators is parallelizing the jobs done on large chunks of data where the sequential access to the individual memory locations is not much necessary during the operation. It is only needed during the initialization of the CAM array where the processor feeds the data as serial. However, even for this purpose, the random-access feature is still not much needed since this copy operation are done in order from the first line until the end. Therefore, the decoder circuit provides over functionality to the overall system which has no additional benefit.

**Figure 4:**
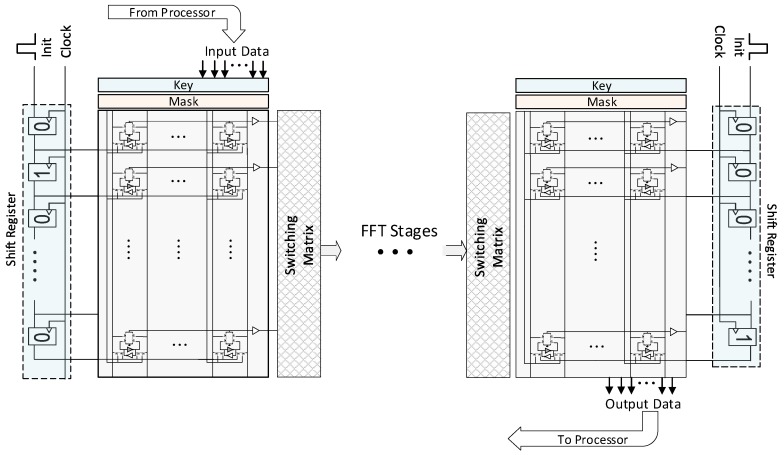
Pipelined in-memory FFT processor architecture.

Instead of using an address decoder, the shift register mechanism is introduced for the sake of area, performance, and energy efficiencies. Figure 4 shows the proposed in-memory FFT architecture explicitly where the costly decoder mechanism is replaced with the shift register-based approach. In this approach, a shift register is placed as vertical to the rows of the AP. The shift register has the same number of registers (flip-flops) as the number of rows in the AP. The outputs of each shift register are connected to the activation input of the corresponding rows. In this case, if the register outputs a logic-1, the row becomes activated while the logic-0 deactivates the corresponding row. The data movement operation from the processor to the AP is performed as follows; First, the processor selects the location of the AP’s columns to which data is written by setting the corresponding mask registers. The processor also asserts the *init pin* of the shift register to initiate the bulk data movement, so that the first register in the shift register becomes logic-1 in the next clock cycle. Therefore, in the first cycle, the first row is activated and ready to be written. At the same time, the outer processor synchronously provides the input data that is written to the selected columns of the first row. In the second cycle, shift register content is shifted by a single bit and the second row is activated and write operation is done for this row. At every time, the activated row by the shift register is written. The processor feeds the data as synchronized with the shift register, so they must be clocked by the same source. In this manner, the write operation for each row continues until reaching to the end row of the AP. To initialize an AP with *n* rows, n+1 cycles are required. In this case, even though the inter-communication between the APs is column-wise through the switching matrix, the communication between the processor and the AP is handled as row-wise but more efficiently. After processing the data in the AP accelerator, the data can be retrieved by the processor in the same manner where the processor reads the data of the activated row from the bit lines as serial. The same shift register can be used for both writing and reading. When compared with the complexity of a decoder circuitry which needs n-1 1-to-2 demultiplexers for n-row CAM, the shift register approach requires n flip-flops only.

### 3.3. Area-Optimized Architecture

**Figure 5:**
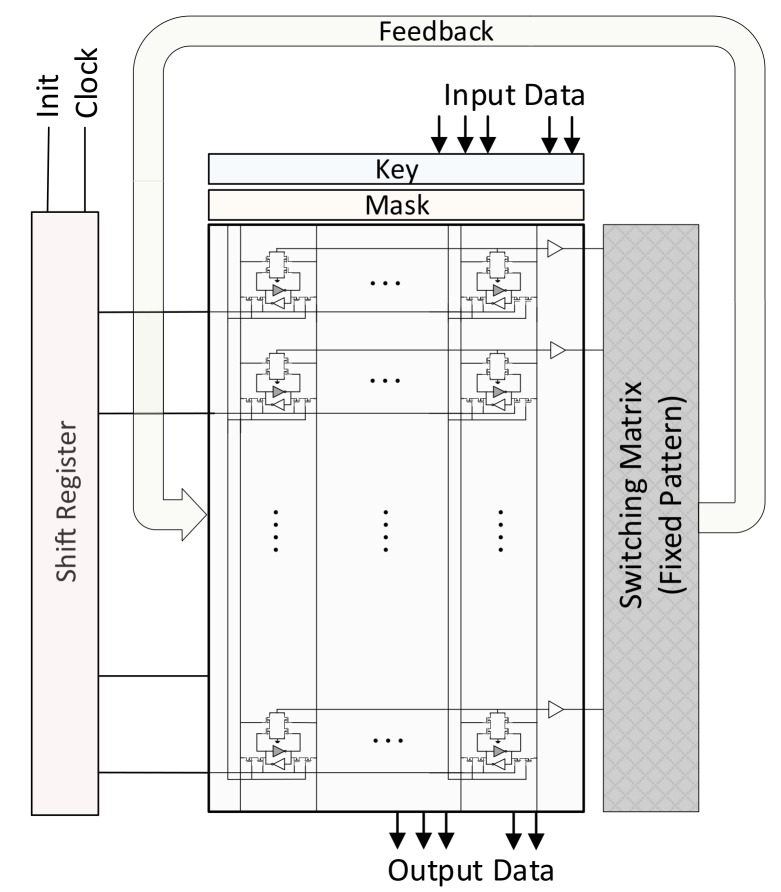
The ultra-area-efficient FFT processor based on singleton’s FFT and feedback.

For the in-memory FFT processors, two different architectures are proposed which are throughput and area-optimized, respectively. The throughput-optimized architecture performs each stage of the FFT in an AP-CAM as shown in Figure 4. The communication patterns between the APs can be fixed since the FFT size is fixed to 1024-point and it is known as a priori. On the other hand, the communication pattern varies with respect to the current stage as seen in Figure 3. Even though this architecture provides high-throughput in-memory FFT, it needs to replacement of AP-CAMs 10 times (i.e., $\log_{2}\left(n\right)$). An area-efficient alternative can be possible through the reconfigurable switching matrix where the results of a single AP stage are feedbacked back to the AP itself (see Figure 5). After completion of a butterfly stage, the reconfigurable switching matrix can be configured according to the next stage. However, this approach requires additional area and control costs. If the number of rows of a CAM array (n) is more than the number of columns (m) in an AP which is generally so since parallelism is obtained as row-wise, the area complexity of a reconfigurable switching matrix (n×n) becomes more than CAM itself (n×m). For instance, to perform a 1024-point FFT on 12-bit data, 132 × 512-bit CAM array is required. On the other hand, it requires a 512 × 512-bit reconfigurable switching matrix. Even the CAM cell size is assumed as 2× of the traditional memory, the switching matrix requires about 1.94× more area. Furthermore, the control over the switching matrix becomes intractable also since every cell must be controlled individually.

**Figure 6:**
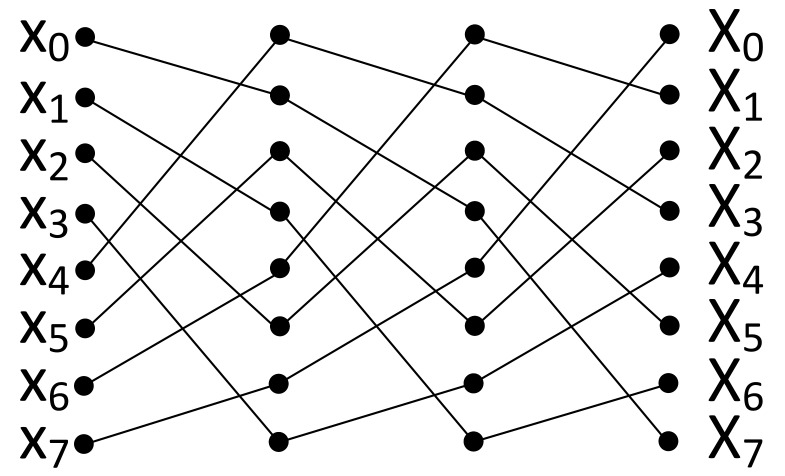
8-point Singleton’s FFT.

There are many algorithmic implementation of FFT (e.g., prime-factor FFT [37], Krukar’s FFT [38], and Bluestein’s FFT [39]) where some of them are optimized for specific input types (e.g., prime sizes, powers of two). Singleton’s FFT [40] is an approach for performing FFT in the same manner and operational complexity as Cooley-Tukey FFT in traditional computers. On the other hand, it provides an incomparable advantage for APs. Even though the traditional FFT requires the change in the communication pattern where each FFT stage requires different input pairs, Singleton’s FFT fixes the pattern of the data flow between the butterfly stages. For the visualization, Figure 6 shows an 8-point FFT using Singleton’s method where the input $x_{i}$ of every step goes into butterfly with input $x_{i+n/2}$ where n is the FFT size. Even though variable computation pattern is not an issue for general-purpose processors or ASICs which always have a random-access memory structure, it provides a vital advantage for parallel in-memory processing systems detailed as follows.

The area-optimized architecture exploits the Singleton’s FFT to fix the inter-communication pattern between the stages. In this way, the whole FFT computation can be performed by using a single FFT stage. If the FFT implementation was the traditional one (i.e., Cooley-Tukey), the switching matrix would have to be reconfigurable. Figure 5 exploits the proposed area-optimized FFT architecture. To move the data from/to processor, a single shift register is used as described above. The switching matrix has a fixed pattern feedbacked to the AP itself so that every FFT stages are performed on the same AP. One drawback of this architecture is that after every computation, the new twiddle factors of the corresponding stage must be loaded to the APs from the outer processor by using the proposed shift register-based data movement approach. On the other hand, the cost of this overhead seems negligible compared with the whole butterfly operation on 1K data.

### 3.4. Dual-Issue Butterfly Operation

**Figure 7:**
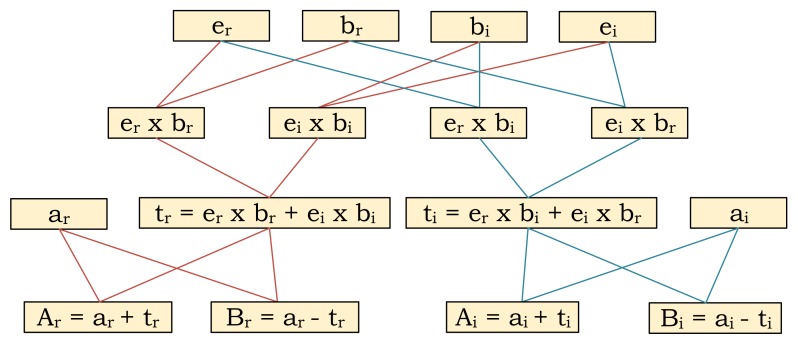
Directed acyclic graph of a butterfly operation.

**Figure 8:**
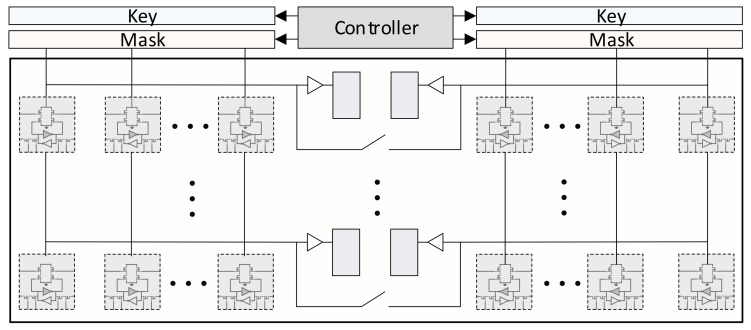
Dual-issue FFT on the AP.

For the further optimization on the performance, the data flow diagram of a single butterfly operation on the AP (i.e., A, B = butterfly(e, a, b)) are inspected. Figure 7 shows the corresponding directed acyclic graph (DAG) of a butterfly operation on the AP where each box corresponds to an operation described inside and the lines show the data dependencies (flow of the data). Since AP performs a complex multiplication operation as four real multiplications, the diagram shows the operations on the real and imaginary parts with subscripts *r* and *i* respectively. At the first insight, it is obvious that the operations show a perfectly symmetric flow. For example, at the beginning while multiplying $e_{r}$ with $b_{r}$, the same multiplication operation of $b_{i}\times e_{i}$ are performed. The set of instructions for performing these operations are the same, therefore can be performed as parallel. At that point, an AP row can be divided into two parts to perform the operations as parallel by adding extra matching circuit. Figure 8 shows the modified architecture for dual-way issue AP. The proposed modification does not require any additional cost to the controller part since the performed operations are identical, so the generated signals for the key and mask registers are exactly the same. At some point, if any operations needs to be performed between the operands on these two parts (e.g., $t_{i}$ computation), the switch between them can be closed and it behaves as a single row. While this modification requires an 10% area overhead to the overall system because of the additional matching circuit, it provides around 1.9× speedup due to the parallel execution of the costly multiplication operations.

## 4. Evaluation

For the evaluation of the proposed in-memory FFT processors (both area-optimized and throughput-optimized), the simulator in [29] are used to perform both system-level and circuit-level (pre-layout) simulations in Matlab and HSPICE, respectively. For the transistor model, the Predictive Technology Models (PTM) [41] is used to simulate high-density memories with 65 nm feature sizes [42]. Even though the used technology is 65 nm, the CAM cells are custom designed to decrease the current leakage and therefore energy consumption since traditional ternary CAM functionality is not needed for APs. The area of the cell design is calculated by referencing the fabricated SRAM and CAM designs in 65 nm [43,44]. The parasitic effects such as the line resistances are taken into account during the circuit simulation to obtain the accurate results [45]. Performance metrics and results are obtained by cross-checking the output of both Matlab and HSPICE simulations. For the sense amplifier, a low-power, sub-ns amplifier design in [46] is employed in the circuit. While comparing the results with the previous studies in the literature, the processors that are in the same category are taken into account. For example, for the data type, only fixed-point FFT processors are compared since it is not fair to compare a fixed-point processor with floating point one.

Table 3 shows the comparison of two in-memory FFT processors with other state-of-the-art FFT processors. The table includes both area-efficient (feedbacked) and throughput-efficient (pipelined) versions of the AP processors indicated as AP (F) and AP (P) respectively. In the AP, all butterfly operations on a CAM are performed simultaneously, so the running time of one stage does not depend on the number of samples if it fits into the memory. On the other hand, the word-length of the FFT operands affects the effective throughput since the operations are done as bitwise. The table shows that the proposed feedbacked in-memory FFT processor has the smallest area. Actually, to store the m-bit FFT operands (i.e., complex numbers) for n-point FFT, 6m×n bits memory is needed. On the other hand, the feedbacked FFT processor performs both storage and computation by using about 11m×n bits memory. When the area of a CAM cell is assumed as 2× of a normal memory cell, this leads to an inference that both computation and storage can be done in around 3.6× of the overall storage area. According to the normalized power results, the proposed processor shows a fair performance. On the other hand, the figure of merit (FOM), an overall evaluation metric of (FFT/s/Energy/Area) shows the best result within the others since the proposed FFT processor provides ultra-area efficiency.

**Figure 9:**
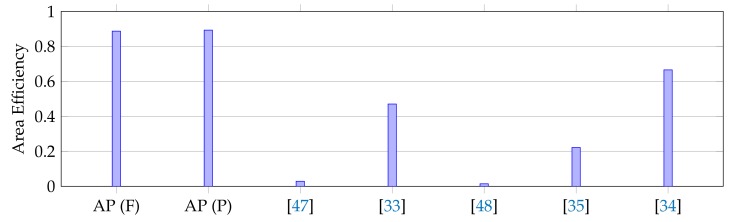
Area efficiencies of FFT processors ($\mathrm{GS}/s/{\mathrm{mm}}^{2}$).

One can put a single multiplier and adder and claim the invention of the smallest FFT processor. Therefore, the smallest area cannot be the sole claim. For this reason, while reporting the results, the GSample/s per area ($\mathrm{GS}/s/{\mathrm{mm}}^{2}$) are provided. Figure 9 proves the overall claim of the study which is proposing an ultra-area-efficient FFT processor. According to the figure, the proposed processors shows the best area efficiency in terms of GSample/s/area when compared with the other processors. In other words, the in-memory FFT processors exhibit the best FFT performance per unit area. The recent study in [34] claims the better normalized throughput per unit area than the state-of-the-art available designs. Beyond this study, the proposed design accomplishes a 33.2% improvement over their reported results.

In some cases, custom FFT processors can be used as directly coupled with an outer data source (sensor, channel, etc.) without any intermediate processor. If there is no outer processor, the coupled system must generate the address while sending the data. A basic counter can be used for this purpose. The proposed methodology of shift register also eliminates this need where the requirement can be fulfilled with a basic shift register. The shift register-based approach can also support multiple writings at the same time (i.e., multi-row activation); however, this is not necessary for the current content. According to the comparison between shift register and address decoder approaches for 1K-FFT processor, the synthesized design on Cadence shows that the shift register consists of fewer flip-flops and logic gates, and hence takes up 25% less area. Furthermore, the shift register is also shown to be more energy efficient which consumes around 0.4× of the address decoder.

**Figure 10:**
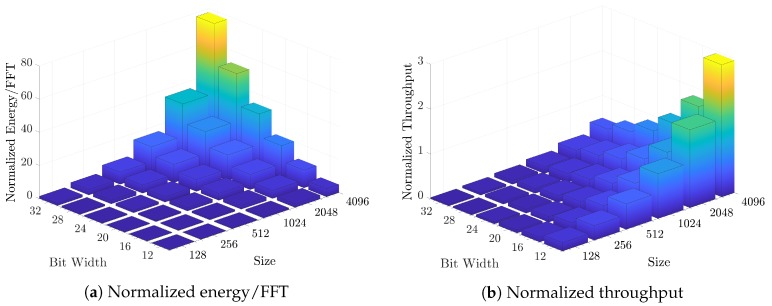
Design space exploration for the area-optimized FFT processor.

**Figure 11:**
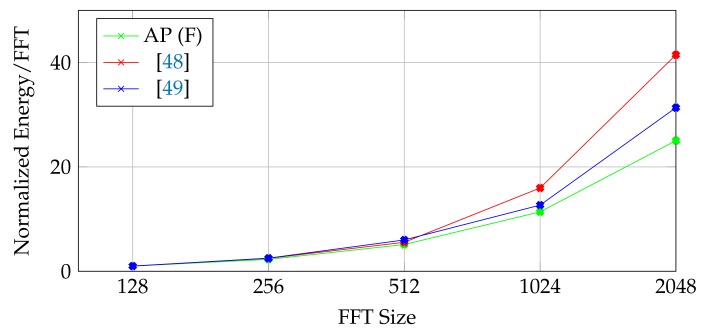
Comparison of normalized Energy/FFT scaling with respect to FFT size.

For a further inspection on the designed architecture, a design space exploration is performed on the architecture with different operand bit widths (12-32 bits) and FFT sizes (128-4K). Figure 10 shows the energy/FFT and throughput results of the area-efficient FFT processors (feedbacked) normalized to 12-bit 1K-point FFT proposed above. Since proposed architecture performs the operations as bitwise, both throughput and energy are highly correlated with it, therefore decreases as bitwidth increases. On the other hand, if the FFT data can fit inside the memory, the throughput of a single butterfly stage increases as O(n). Overall, FFT throughput depends on the total number of stages as well which is formulated as $O(\log_{2}n)$. In overall, the normalized throughput with respect to FFT size changes by $O(n/\log_{2}n)$. In traditional FFT architectures, the throughput of a single butterfly stage decreases as FFT size increases since it needs to use the available resources sequentially, therefore, overall throughput changes by $O(1/\left(n\times \log_{2}n\right))$. Figure 11 shows the energy/FFT results for both the proposed FFT and the architectures from [48,49] where the FFT size changes between 128–2048 points. The architectures in [48,49] can be configured to perform 128, 256, 512, 1024, or 2048-point FFTs. The result demonstrates that proposed AP-based FFT shows better scaling in terms of energy/FFT with respect to increasing FFT sizes. Since the need for higher point FFT increases in the domains such as MRI which also requires parallel computation on the data coming from many receivers [50], the in-memory FFT architecture can propose an efficient solution together with the high-speed data placement through the proposed shift register-based approach.

**Figure 12:**
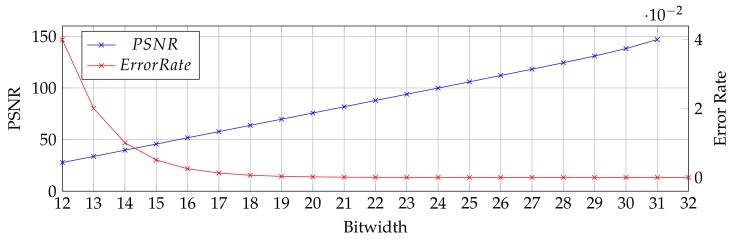
Bitwidth vs. average PSNR and error rate of 1024-point FFT.

Even though the proposed FFT processor achieves a great deal of area efficiency due to the dense structure of the memory arrays, another paradigm that can be beneficial on this architecture is approximate computing. Approximate computing is a popular computing paradigm that relaxes the correctness constraints of a system for the sake of energy and performance improvement [51,52]. The paradigm can be applied to the error-tolerant applications. APs facilitate the approximate computing inherently since the operations are performed bit-by-bit basis [28]. As an example case, the proposed architecture can be evaluated for communication applications in which the bitwidth of the FFT processor can be adjusted dynamically during the run time concerning the estimated channel signal-to-noise ratio (SNR), aiming at achieving the desired performance at a reduced energy consumption [32]. Figure 12 shows an example case for 1K FFT where the change in average peak signal-to-noise ratio (PSNR) and error rate with respect to the bitwidth are shown where the reference is 32-bit FFT. When interpreted with Figure 10 where the normalized energy and throughput results are presented with respect to bitwidth, the approximate in-memory FFT can be performed dynamically by adjusting the bitwidth during runtime to obtain the optimum energy consumption together with the required throughput.

## 5. Conclusions

In this study, an ultra-area-efficient FFT processor through the in-memory associative processor is introduced. The proposed processor performs FFT directly inside the memory. For better communication with the external systems, the traditional accelerator architecture is improved by proposing a better data moving mechanism specific to the AP-based accelerators. Furthermore, the study introduces a dual-way associative processing methodology to perform the symmetric tasks of the butterfly operation at nearly 2× speed without any cost to the controller. The proposed design has the smallest area occupancy reported until now. The efficiency of the proposed architecture is proven by comparing it with the state-of-the-art FFT processors in terms of performance, power, and area. Beyond the smallest reported area, the proposed processor achieves the best area efficiency (normalized throughput per area) within its own class of FFT processors. It means that the proposed architecture delivers the best performance in a given area.

## Figures and Tables

**Table 1:** Computation types with respect to memory.

Computation Type	Data Location	Functionality Location	Bandwidth Constraint
Traditional	Separate IC	Processor	Inter-chip Bus
Near-memory	Same IC	Processor	In-chip Bus
In-memory	Same IC	Memory	Memory Capacity

**Table 2:** LUTs for addition and subtraction.

			Addition			Subtraction		
*Compare*			*Write*			*Write*		
Cr/Br	B	A	Cr	B	Comment	Br	B	Comment
0	0	0	0	0	NC	0	0	NC
0	0	1	0	1	2ndPass	1	1	1stPass
0	1	0	0	1	NC	0	1	NC
0	1	1	1	0	1stPass	0	0	2ndPass
1	0	0	0	1	3rdPass	1	1	4thPass
1	0	1	1	0	NC	1	0	NC
1	1	0	1	0	4thPass	0	0	3rdPass
1	1	1	1	1	NC	1	1	NC

**Table 3:** Comparison of FFT Processors without normalization.

Specification	AP (F)	AP (P)	[47]	[33]	[48]	[35]	[34]
FFT Size (N)	1024	1024	1024	256	2048	1024	4096
Technology	65 nm	65 nm	65 nm	90 nm	65 nm	65 nm	65 nm
$V_{\mathrm{dd}}$	0.45 V	0.45 V	0.27 V	1 V	0.45 V	0.6 V	1.2 V
Word-length	12-bit	12-bit	16-bit	10-bit	12-bit	32-bit *	14-bit
Area	${0.099\;\mathrm{mm}}^{2}$	${0.99\;\mathrm{mm}}^{2}$	${8.29\;\mathrm{mm}}^{2}$	${5.1\;\mathrm{mm}}^{2}$	${1.37\;\mathrm{mm}}^{2}$	${3.6\;\mathrm{mm}}^{2}$	${1.46\;\mathrm{mm}}^{2}$
Power	12 mW	123 mW	4.15 mW	165 mW	1.01 mW	60.3 mW	68.6 mW
Throughput/Area ($\mathrm{GS}/s/{\mathrm{mm}}^{2}$)	0.89	0.89	0.03	0.47	0.015	0.22	0.67
FOM (FFT/Energy/Area)	70.4	7.09	6.82	15.3	7.04	3.60	2.37

* The bitwidth of the architecture is variable over the FFT stages and the maximum one is 32-bit.

## References

[1] Mutlu O., Ghose S., Gómez-Luna J., Ausavarungnirun R. (2019). Processing data where it makes sense: Enabling in-memory computation. Microprocess. Microsyst..

[2] (2018). Big data needs a hardware revolution. Nature.

[3] Dally W.J. Challenges for Future Computing Systems. Proceedings of the 2015 Amsterdam Conference.

[4] Ghose S., Hsieh K., Boroumand A., Ausavarungnirun R., Mutlu O. (2018). Enabling the Adoption of Processing-in-Memory: Challenges, Mechanisms, Future Research Directions. arXiv.

[5] Kozyrakis C.E., Perissakis S., Patterson D., Anderson T., Asanovic K., Cardwell N., Fromm R., Golbus J., Gribstad B., Keeton K. (1997). Scalable processors in the billion-transistor era: IRAM. Computer.

[6] Gokhale M., Lloyd S., Macaraeg C. (2015). Hybrid Memory Cube Performance Characterization on Data-centric Workloads. Proceedings of the 5th Workshop on Irregular Applications: Architectures and Algorithms.

[7] Ghose S., Hsieh K., Boroumand A., Ausavarungnirun R., Mutlu O., Topaloglu R.O., Wong H.S.P. (2019). The Processing-in-Memory Paradigm: Mechanisms to Enable Adoption. Beyond-CMOS Technologies for Next Generation Computer Design.

[8] Kanev S., Darago J.P., Hazelwood K., Ranganathan P., Moseley T., Wei G.Y., Brooks D. (2015). Profiling a Warehouse-scale Computer. Proceedings of the 42nd Annual International Symposium on Computer Architecture.

[9] Seshadri V., Kim Y., Fallin C., Lee D., Ausavarungnirun R., Pekhimenko G., Luo Y., Mutlu O., Gibbons P.B., Kozuch M.A. RowClone: Fast and energy-efficient in-DRAM bulk data copy and initialization. Proceedings of the 2013 46th Annual IEEE/ACM International Symposium on Microarchitecture (MICRO).

[10] Mittal S. (2018). A Survey of ReRAM-Based Architectures for Processing-In-Memory and Neural Networks. Mach. Learn. Knowl. Extr..

[11] Ielmini D., Wong H.S.P. (2018). In-memory computing with resistive switching devices. Nat. Electron..

[12] Li S., Xu C., Zou Q., Zhao J., Lu Y., Xie Y. Pinatubo: A Processing-in-memory Architecture for Bulk Bitwise Operations in Emerging Non-volatile Memories. Proceedings of the 53rd Annual Design Automation Conference.

[13] Sim J., Imani M., Choi W., Kim Y., Rosing T. LUPIS: Latch-up based ultra efficient processing in-memory system. Proceedings of the 2018 19th International Symposium on Quality Electronic Design (ISQED).

[14] Chen B., Cai F., Zhou J., Ma W., Sheridan P., Lu W.D. Efficient in-memory computing architecture based on crossbar arrays. Proceedings of the 2015 IEEE International Electron Devices Meeting (IEDM).

[15] Imani M., Gupta S., Rosing T. (2017). Ultra-Efficient Processing In-Memory for Data Intensive Applications. Proceedings of the 54th Annual Design Automation Conference 2017.

[16] Chi P., Li S., Xu C., Zhang T., Zhao J., Liu Y., Wang Y., Xie Y. PRIME: A Novel Processing-in-Memory Architecture for Neural Network Computation in ReRAM-Based Main Memory. Proceedings of the 2016 ACM/IEEE 43rd Annual International Symposium on Computer Architecture (ISCA).

[17] Stone H.S. (1970). A Logic-in-Memory Computer. IEEE Trans. Comput..

[18] Santoro G., Turvani G., Graziano M. (2019). New Logic-In-Memory Paradigms: An Architectural and Technological Perspective. Micromachines.

[19] Cofano M., Vacca M., Santoro G., Causapruno G., Turvani G., Graziano M. (2019). Exploiting the Logic-In-Memory paradigm for speeding-up data-intensive algorithms. Integration.

[20] Chua L. (1971). Memristor-The missing circuit element. IEEE Trans. Circuit Theory.

[21] Apalkov D., Khvalkovskiy A., Watts S., Nikitin V., Tang X., Lottis D., Moon K., Luo X., Chen E., Ong A. (2013). Spin-transfer Torque Magnetic Random Access Memory (STT-MRAM). J. Emerg. Technol. Comput. Syst..

[22] Hennig J., Nauerth A., Friedburg H. (1986). RARE imaging: A fast imaging method for clinical MR. Magn. Reson. Med..

[23] Li L., Wyrwicz A.M. (2018). Parallel 2D FFT implementation on FPGA suitable for real-time MR image processing. Rev. Sci. Instrum..

[24] Shi L., Andronesi O., Hassanieh H., Ghazi B., Katabi D., Adalsteinsson E. Mrs sparse-fft: Reducing acquisition time and artifacts for in vivo 2d correlation spectroscopy. Proceedings of the International Society for Magnetic Resonance in Medicine Annual Meeting and Exhibition (ISMRM’13).

[25] Potter J.L. (1991). Associative Computing: A Programming Paradigm for Massively Parallel Computers.

[26] Foster C.C. (1976). Content Addressable Parallel Processors.

[27] Yavits L., Morad A., Ginosar R. (2015). Computer Architecture with Associative Processor Replacing Last-Level Cache and SIMD Accelerator. IEEE Trans. Comput..

[28] Yantir H.E., Eltawil A.M., Kurdahi F.J. (2017). Approximate Memristive In-memory Computing. ACM Trans. Embed. Comput. Syst..

[29] Yantır H.E., Eltawil A.M., Kurdahi F.J. (2018). A Hybrid Approximate Computing Approach for Associative In-Memory Processors. IEEE J. Emerg. Sel. Top. Circuits Syst..

[30] Fourier J., Grattan-Guinness I., Cooke R., Corry L., Crépel P., Guicciardini N. (2005). Chapter 26—Joseph Fourier, Théorie analytique de la chaleur (1822). Landmark Writings in Western Mathematics 1640–1940.

[31] Cooley J., Tukey J. (1965). An Algorithm for the Machine Calculation of Complex Fourier Series. Math. Comput..

[32] Abdelaal R.A., Yantır H.E., Eltawil A.M., Kurdahi F.J. (2019). Power Performance Tradeoffs Using Adaptive Bit Width Adjustments on Resistive Associative Processors. IEEE Trans. Circuits Syst. Regul. Pap..

[33] Chen Y., Lin Y.W., Tsao Y.C., Lee C.Y. (2008). A 2.4-Gsample/s DVFS FFT Processor for MIMO OFDM Communication Systems. IEEE J. -Solid-State Circuits.

[34] Liu S., Liu D. (2018). A High-Flexible Low-Latency Memory-Based FFT Processor for 4G, WLAN, and Future 5G. IEEE Trans. Very Large Scale Integr. Syst..

[35] Ba N.L., Kim T.T. (2018). An Area Efficient 1024-Point Low Power Radix-22FFT Processor With Feed-Forward Multiple Delay Commutators. IEEE Trans. Circuits Syst. I Regul. Pap..

[36] Guo Q., Guo X., Patel R., Ipek E., Friedman E.G. (2013). AC-DIMM: Associative Computing with STT-MRAM. SIGARCH Comput. Archit. News.

[37] Good I.J. (1958). The Interaction Algorithm and Practical Fourier Analysis. J. R. Stat. Soc. Ser. B.

[38] Rader C.M. (1968). Discrete Fourier transforms when the number of data samples is prime. Proc. IEEE.

[39] Bluestein L. (1970). A linear filtering approach to the computation of discrete Fourier transform. IEEE Trans. Audio Electroacoust..

[40] Singleton R. (1967). A method for computing the fast Fourier transform with auxiliary memory and limited high-speed storage. IEEE Trans. Audio Electroacoust..

[41] Arizona State University (2011). Predictive Technology Model (PTM).

[42] Sinha S., Yeric G., Chandra V., Cline B., Cao Y. Exploring sub-20nm FinFET design with Predictive Technology Models. Proceedings of the DAC Design Automation Conference 2012.

[43] Zhang K., Bhattacharya U., Chen Z., Hamzaoglu F., Murray D., Vallepalli N., Wang Y., Zheng B., Bohr M. SRAM design on 65nm CMOS technology with integrated leakage reduction scheme. Proceedings of the 2004 Symposium on VLSI Circuits. Digest of Technical Papers.

[44] Hayashi I., Amano T., Watanabe N., Yano Y., Kuroda Y., Shirata M., Dosaka K., Nii K., Noda H., Kawai H. (2013). A 250-MHz 18-Mb Full Ternary CAM With Low-Voltage Matchline Sensing Scheme in 65-nm CMOS. IEEE J. -Solid-State Circuits.

[45] Wilson L. (2013). International technology roadmap for semiconductors (ITRS).

[46] Schinkel D., Mensink E., Klumperink E., van Tuijl E., Nauta B. A Double-Tail Latch-Type Voltage Sense Amplifier with 18ps Setup+Hold Time. Proceedings of the 2007 IEEE International Solid-State Circuits Conference.

[47] Seok M., Jeon D., Chakrabarti C., Blaauw D., Sylvester D. A 0.27 V 30 MHz 17.7 nJ/transform 1024-pt complex FFT core with super-pipelining. Proceedings of the 2011 IEEE International Solid-State Circuits Conference Digest of Technical Papers (ISSCC).

[48] Yang C., Yu T., Markovic D. (2012). Power and Area Minimization of Reconfigurable FFT Processors: A 3GPP-LTE Example. IEEE J. -Solid-State Circuits.

[49] Guichang Z., Fan X., Willson A.N. (2006). A power-scalable reconfigurable FFT/IFFT IC based on a multi-processor ring. IEEE J. -Solid-State Circuits.

[50] McDougall M.P., Wright S.M. (2005). 64-channel array coil for single echo acquisition magnetic resonance imaging. Magn. Reson. Med..

[51] Mittal S. (2016). A Survey of Techniques for Approximate Computing. ACM Comput. Surv..

[52] Agrawal A., Choi J., Gopalakrishnan K., Gupta S., Nair R., Oh J., Prener D.A., Shukla S., Srinivasan V., Sura Z. Approximate computing: Challenges and opportunities. Proceedings of the 2016 IEEE International Conference on Rebooting Computing (ICRC).

